# Vector competence of Belgian *Anopheles plumbeus* mosquitoes for West Nile virus under different temperature conditions

**DOI:** 10.1186/s13071-026-07346-9

**Published:** 2026-04-03

**Authors:** Sara Goossens, Charlotte Sohier, Nick De Regge

**Affiliations:** Exotic and Vector-Borne Diseases, Sciensano, Brussels, Belgium

**Keywords:** West Nile virus, Vector competence, *Anopheles plumbeus*, Belgium

## Abstract

**Background:**

Belgium will likely be confronted with the introduction of West Nile virus (WNV) in the near future, as its presence in neighboring countries has been documented, and the high abundance of *Culex pipiens*, the main vector of this virus, has been documented in Belgium as well. Other mosquito species, such as *Anopheles plumbeus*, could also play a role in disease transmission. *Anopheles plumbeus* is a tree hole-breeding species whose habitat is expanding to urban sites, thus increasing its contact with humans. Only limited data are available on the vector competence of this species for arboviruses, let alone WNV. Such knowledge is important with respect to risk assessments and a preparedness plan in case a WNV introduction in Belgium takes place.

**Methods:**

A vector competence study with field-collected Belgian *Anopheles plumbeus* mosquitoes for WNV (lineage 1) was performed under different temperature conditions: a constant 25 °C, 25/20 °C, and 25/15 °C day/night temperature gradient.

**Results:**

At 14 days post-blood-feeding, the different temperature conditions did not impact the infection rate, ranging between 24% and 29%, nor the viral load in the gut, indicating a similar level of viral replication. Interestingly, statistically significant differences in virus dissemination from the gut to secondary tissues were observed between the different conditions. At a constant temperature of 25 °C, WNV was detected via quantitative reverse transcription polymerase chain reaction (RT-qPCR) in the saliva of one mosquito, resulting in a transmission efficiency of 1.6%. In contrast, no WNV was detected in the saliva under gradient conditions. WNV could not be isolated from the RT-qPCR positive saliva sample.

**Conclusions:**

Since no proof could be found of infectious WNV being present in the saliva of *Anopheles plumbeus* under any temperature condition, this species cannot be considered a competent vector on the basis of our results. As also only a low viral RNA load was detected by RT-qPCR in one saliva sample at the 25 °C condition, we conclude that *Anopheles plumbeus* will probably play no role in WNV transmission in Belgium, especially under temperature conditions that more closely reflect the current Belgian climate (25/15 °C) or what could occur in the future (25/20 °C) during the vector season.

**Graphical Abstract:**

**Figure:**
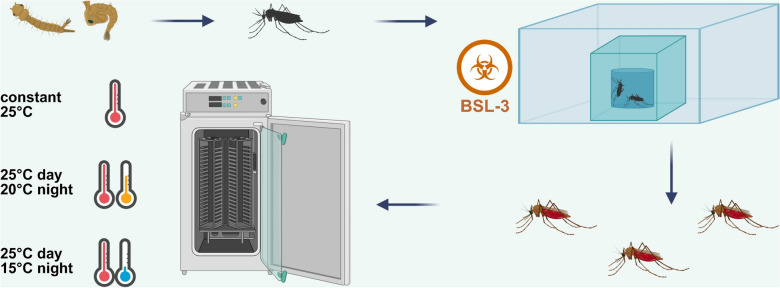

**Supplementary Information:**

The online version contains supplementary material available at 10.1186/s13071-026-07346-9.

## Background

West Nile virus (WNV) is an emerging arbovirus that has spread rapidly across large parts of Europe during the past two decades. It is a mosquito-borne virus belonging to the Japanese encephalitis virus (JEV) serocomplex within the *Orthoflavivirus* genus of the *Flaviviridae* family. The viral genome is a positive-sense, single-stranded RNA molecule of ~ 11 kilobases that is translated into three structural (premembrane, envelope, and nucleocapsid) and seven nonstructural (NS1, NS2A, NS2B, NS3, NS4A, NS4B, and NS5) proteins [1]. There are up to nine known genetic lineages of WNV, of which lineages 1a and 2 are the most prevalent in Europe. Owing to their widespread presence and high virulence in humans, these lineages position WNV as a major public health concern in Europe [1, 2].

WNV is considered the globally most widespread arbovirus and the leading cause of arboviral encephalitis worldwide [3]. In Europe, WNV has become an increasing threat to human and animal health because of its large spread over the continent and the multitude of outbreaks that have occurred over the past 20 years. The virus was able to spread rapidly, and is now considered endemic in several parts of southern and southeastern Europe [4]. Although WNV has been reported in our neighboring countries France [5], Germany [6], and the Netherlands [7, 8], it has never been detected in Belgium to date. However, the presence of competent mosquito vectors and ongoing viral circulation in adjacent regions underscore the risk for a potential WNV introduction and spread in Belgium.

WNV is transmitted via an enzootic cycle between ornitophagic mosquitoes and birds, which serve as amplification hosts, as they develop viremia that is high enough to infect a “naïve” feeding mosquito. Humans and horses can also become infected with WNV after the bite of an infected mosquito, but they do not develop sufficiently high viremia to support further transmission. They are therefore called dead-end hosts. Although they do not contribute to further transmission, dead-end hosts can still develop severe clinical symptoms [9]. Approximately 20–30% of human WNV infections are symptomatic. The most common clinical image is a mild febrile illness, which is characterized by unspecific flu-like symptoms such as fatigue, headaches, arthralgia and myalgia. This clinical picture is referred to as West Nile fever. In an estimated 1% of cases, the infection evolves toward West Nile neuroinvasive disease, which is associated with a 10% mortality rate, and among survivors persistent neurological complications occur in 20–40% of cases [4, 9, 10]. Treatment options for WNV infection are scarce, as currently only equine vaccines for WNV are available, making vector control an important pillar in disease prevention [11].

The main vectors for WNV are *Culex (Cx.)* spp. mosquitoes, belonging to the *Culex pipiens* L. complex, which includes *Cx. pipiens*, the most abundant mosquito species in Belgium [3]. Additionally, several *Aedes (Ae.)* spp. mosquitoes are known vectors for the virus, such as *Ae. vexans*, of which vector competence has been shown not only to approach that of *Cx. pipiens* [12], but was also found to be positive for WNV in nature [13]. The vector competence of European *Ae. japonicus* [14, 15] and *Ae. albopictus* [16, 17], two invasive species also found in Belgium [18], has been proven in a laboratory setting. Nevertheless, only US studies have reported the detection of WNV-positive *Ae. japonicus* and *Ae. albopictus* in the field to date [19, 20], whereas no such data exist for Europe.

Many other mosquito species exist, which have not yet been evaluated for their role in the transmission of mosquito-borne pathogens. One of them is *Anopheles (An.) plumbeus* (Stephens 1828), a Belgian native mosquito species that was never considered a vector species of importance due to its woodland ecology as a tree hole-breeding species. However, in recent years, the breeding sites of *An. plumbeus* have evolved into more human-made environments, increasing their potential as bridge vectors for mosquito-borne pathogens due to their increased contact with humans [21, 22]. Furthermore, *An. plumbeus* is a nuisance species with aggressive biting behavior toward humans and birds, which further increases its potential as a bridge vector for the transmission of pathogens between birds, the reservoir host of WNV, and humans [21]. *Anopheles plumbeus* is a known vector for malaria [23, 24], but only little information exists regarding its vector competence for WNV. A study from the 1960s reported *An. plumbeus* as a competent vector for WNV [25], whereas a recent study found *An. plumbeus* to be an incompetent vector for WNV [26].

With respect to the potential risk of a WNV introduction in Belgium, we performed a vector competence study with field-collected Belgian *An. plumbeus* for WNV. In addition to the assessment at a constant incubation temperature, as is mostly done, we also investigated whether the introduction of day/night temperature gradients, which are more representative of the actual conditions during Belgian summers, would influence vector competence. Therefore, this vector competence study was performed at a constant 25 °C temperature, at a 25/15 °C day/night gradient, which reflects current Belgian summers, and at a 25/20 °C day/night gradient, which reflects a possible scenario of future Belgian summers with higher night temperatures in the context of global warming [27, 28].

## Methods

### Mosquito collection and rearing

Larvae were field-collected during the summers of 2023 and 2024 in De Vortebossen, Ruiselede, Belgium (51°04′15.0’’N, 3°22′00.1’’E), a collection site known to produce solely *An. plumbeus*, as confirmed by adult identifications via the MosKeyTool software (version 2.3.2; https://www.onehealthsecure.com/entomology-tools-0/moskeytool). Larvae were collected in plastic containers with rainwater from the collection site and were provided a mix of koifood (Good Pearls Koi, Tom & Co), rabbit food (CuniAdult Complete, Versele-Laga), and liver powder (QenQ baitproducts) three times a week at a 1:1:1 ratio at a concentration of 200 mg/L. Larvae and adults were kept at 25 °C, 80% relative humidity with a 16:8 h light–dark cycle. A 10% sugar solution made of dissolved granulated sugar (Delhaize) in tap water was offered ad libitum as a food source to emerged adults.

### Virus production and titration

A ninth passage of WNV lineage 1 (strain Israel 98, GenBank: NC_009942.1) was used. This stock was produced on 80% confluent Vero cells, maintained at 37 °C in Dulbecco’s modified Eagle’s medium (DMEM, Thermo Fisher), and supplemented with 1% antibiotics (50 mg/mL gentamicin, Thermo Fisher; Antibiotic Antimycotic Solution 100, Sigma‒Aldrich) and 10% fetal bovine serum (FBS, Merck Life Science). At 72 h post-inoculation, the viral stocks were placed at −80 °C, and the supernatant was collected after three freeze‒thaw cycles. To determine the titer of the virus stock, titrations were performed on 96-well plates seeded with 80% confluent Vero cells. Tenfold serial dilutions were prepared from the viral stock in DMEM supplemented with 0.2% gentamycin, 0.2% amphotericin B (250 µg/mL, Thermo Fisher), and 2% FBS. Eight replicates of 100 µL of each dilution were added to the Vero cells grown in 96-well plates and negative (DMEM) and positive (undiluted WNV stock) controls were included. Cells were incubated with the inoculum at 37 °C. After 27 h of incubation, the inoculum was removed from the cells, and cells were washed with phosphate-buffered saline 1x (PBS, Merck Life Science). The cells were fixed by adding methanol absolute (kept at −20 °C; Thermo Fisher) to the cells for 20 min. After removal of the methanol, the plates were placed with an open lid at −20 °C for a minimum of 24 h to evaporate any remaining methanol. Thereafter, virus detection was achieved via immunofluorescence staining with a primary NS1 antibody (viral West Nile virus NS1 Antibody, Bio-Techne, UK, Catalog #MAB290721) and a secondary Alexa Fluor^®^ 488 anti-mouse IgG2b Antibody (BioLegend, Catalog #406,718). Titration showed the titer of the used virus stock to be 10^6.2^ TCID50/mL (50% median tissue culture infectious dose per mL).

### Oral infection and incubation of mosquitoes

The 3–7-day-old F0 generation *An. plumbeus* mosquitoes were deprived of any food source for 24 h prior to infection. Subsequently, the mosquitoes were transported to the BSL-3 facilities, where blood-feeding was performed for 1 h via the Hemotek with pig intestine as a membrane. One Hemotek feeder with a volume of 1 mL was used per feeding event. The blood meal consisted of a 1:2 ratio of WNV stock and fresh chicken blood collected at the slaughterhouse supplemented with adenosine triphosphate (ATP; Merck Life Science; final concentration 5 µM), corresponding to a titer of 10^5.7^ TCID50/mL. After blood-feeding, blood-fed females were selected, transferred to a Bugdorm mosquito cage, and kept at a constant 25 °C, 25/15 °C day/night temperature gradient or 25/20 °C day/night temperature gradient (with gradual increases/decreases in temperature and light intensity over 4 h) at 80% relative humidity and a 16:8 h light–dark cycle. A 10% sucrose solution was provided ad libitum during the 14-day incubation period. After blood-feeding and before releasing them in Hemotek cages, blood-fed females were morphologically checked to have maxillary palps, which are almost as long as the proboscis and long-haired “brushy” antennae, being specific traits for Anopheles spp. and *An. plumbeus*, respectively.

### Mosquito salivation and dissection

At 14 days post-infection (dpi), the surviving WNV-exposed mosquitoes were cold anesthetized at 4 °C and kept on ice during sample collection. Legs and wings were first collected to prevent the escape of infected mosquitoes. For saliva collection, mosquitoes (without legs and wings) were fixed to a glass slide with double-sided adhesive tape, and their proboscis was inserted into a 10 µL pipette tip filled with 5 µL of FBS, which was held in place using modeling clay [29]. Mosquitoes were allowed to salivate for 20 min. After 20 min, the content of the tip was transferred into an Eppendorf tube containing 45 µL DMEM with 2% antibiotics/antimycotics and 2% FBS, and used for the determination of transmission rate. The mosquito head was collected together with the previously dissected legs and wings (further described as secondary tissues) in an Eppendorf tube containing 500 µL DMEM supplemented with 2% antibiotics/antimycotics to determine dissemination rate. The remaining thorax and abdomen of the mosquito were collected into a third Eppendorf tube containing the same DMEM/antibiotics mixture as the secondary tissues for determination of the infection rate [40]. In this study the infection rate is defined as the ratio of the number of WNV-positive abdomen samples in quantitative reverse transcription polymerase chain reaction (RT-qPCR) at 14 days post-blood-feeding to the total number of female mosquitoes that took a blood meal and survived the 14-day incubation period. The dissemination rate is defined as the ratio of mosquitoes that were WNV RT-qPCR positive in secondary tissues to the total number of mosquitoes that took an infectious blood meal and survived the 14-day incubation period. Lastly, the transmission efficiency is defined as the ratio of the number of WNV-RT-qPCR-positive saliva samples to the total number of mosquitoes that took a blood meal and survived the 14-day incubation period.

### WNV detection

#### RT-qPCR analysis

Silicon-carbide beads (1,0 mm diameter, Biospec) were added to the mosquito thorax–abdomen and to the secondary tissue samples that were kept in a DMEM/antibiotics solution, and homogenized via a Tissuelyser (Tissuelyser II, Qiagen,). RNA was extracted via the QIAamp Viral RNA Mini Kit (Qiagen) according to the manufacturer’s protocol. As the procedure is optimized for 140 µL input volumes, saliva samples were adjusted to 140 µL (25 µL sample + 115 µL nuclease-free water). The extracted RNA was used directly for RT-qPCR analysis or stored at −80 °C until further use. The one-step RT-qPCR mixture for WNV detection contained 12.5 µL AgPath-ID^™^ One-Step PCR buffer (2x), 2 µL primer/probe mixture, 1 µL of reverse transcriptase, 4.5 µL of nuclease-free water, and 5 µL of the RNA sample. The final concentrations of the WNV NS2A primers (forward: 5ʹ—CCTTTTCAGTTGGGCCTTCTG—3ʹ and reverse: 5ʹ—ATCTTGGCYGTCCACCTCTTG—3ʹ) and probe (5ʹ—FAM/TTCTTGGCC/ ZEN/ACCCAGGAGGTC/IABkFQ—3ʹ) [30] were 1 µM and 400 nM, respectively. A negative control (nuclease-free water) was added to each run, as well as a positive control (RNA extract of a WNV virus stock dilution with a cycle threshold value [CT] value ~23). The samples were run on a LightCycler 480 according to the following temperature program: 45 °C for 10 min and 95 °C for 10 min, followed by 45 cycles at 95 °C for 15 s and 60 °C for 45 s. Samples with a CT value < 40 and curves that showed exponential amplification were considered positive. Samples with a CT value < 40 lacking a characteristic exponential amplification curve were considered doubtful and retested in a two-step RT-qPCR for confirmation, which is explained in detail further below. Each infection and dissemination sample was also tested for amplification of beta-actin, a housekeeping gene, as an internal control. A similar one-step RT-qPCR mixture was prepared for the detection of beta-actin with 600 nM universal beta-actin primers (forward: 5ʹ—CAGCACAATGAAGATCAAGATC ATC—3ʹ and reverse: 5ʹ—CGGACTCATCGTACTCCTGCTT—3ʹ) and 400 nM probe (5ʹ—HEX/TCGCTGTCC/ZEN/ACCTTCCAGCAGATGT/IABkFQ—3ʹ) [31] in the mixture. A synthetic RNA construct was added to the saliva samples as an external extraction control (EEC), and successful extraction was assessed by amplification of the EEC via RT-qPCR via a similar one-step RT-qPCR mixture as described for WNV but with the following primer/probe mixture: 1 µM of EEC forward: 5ʹ— ATAAATAATCTGACGTTTGTAATGTCCGCTC—3ʹ and reverse 5ʹ—ATAAATAATCTCCAGTTGCTACCGATTTTACATA—3’ primers and 200 nM probe (5ʹ—TexRd‒XN/TTTGTACCACCTCCCACCGACCATC/IAbRQSp—3ʹ) [32]. The samples whose results for WNV in the AgPath One-Step PCR were doubtful (CT value < 40, but without a corresponding amplification curve) were retested in a two-step RT-qPCR for confirmation. The two-step RT-qPCR provides better sigmoidal amplification curves for low positive samples. Doubtful samples positive in the two-step RT-qPCR were considered positive and their CT value obtained in the one-step RT-qPCR was used for further analysis. Samples negative in the two-step RT-qPCR were considered negative for further analysis. The following mixture was used for reverse transcription: 4 µL first strand buffer (5x, Invitrogen), 2 µL DTT (Invitrogen), 2 µL dNTPs at a concentration of 5 mM (Invitrogen), 4 µL random hexamers at a concentration of 10 µM (Invitrogen), 0.25 µL RNase inhibitor RNase OUT (Invitrogen), 0.5 µL reverse transcriptase (Invitrogen), 3.25 µL RNase-free water, and 4 µL RNA sample. Reverse transcription was performed according to the following temperature protocol: 37 °C for 1 h, followed by 10 min at 65 °C. After reverse transcription, the following qPCR mixture was used: 10 µL LightCycler480 probe master mixture (Roche), 2 µL primer/probe mixture, 0.32 µL MgCl_2_, 5.18 µL nuclease-free water, and 2.5 µL cDNA. This qPCR was run on a LightCycler480 according to the following protocol: 5 min at 95 °C, followed by 45 cycles at 95 °C for 15 s and 60 °C for 45 s. Two-step PCR results were considered positive when CT values ≤ 40 with an exponential amplification curve were obtained.

A standard curve was generated by testing tenfold serial dilutions (performed in threefold) of a WNV stock in the one-step RT-qPCR. Via this standard curve and by taking the homogenization volume of the collected samples into account, the CT value per sample was converted in the equivalent viral RNA load expressed in TCID50 per sample.

#### Virus isolation

Abdomens, secondary tissue samples, and saliva found positive by RT-qPCR were tested in virus isolation to assess the presence of infectious virus. After being thawed and preheated to 37 °C, 50 µL of the homogenate of abdomens and secondary tissue samples and 25 µL of the collected saliva supplemented with 25 µL DMEM was inoculated into a well in a 96-well plate containing 80% confluent Vero cells. After inoculation, the cells were incubated at 37 °C for 2 h before 100 µL of DMEM containing 2% antibiotics/antimycotics and 2% FBS was added. The plates were then incubated for 7 days at 37 °C. At 7 dpi, a second passage on Vero cells was performed by transferring 50 µL of the supernatant to a new 96-well plate, and the protocol described above was repeated. Fixation and staining was performed as described above in the paragraph about virus production and titration.

### Statistical analysis

A linear regression analysis was implemented to assess whether an association existed between the number of mosquitoes exposed to a blood meal and the feeding rate. The Shapiro‒Wilk test was performed on all the data to determine whether the data were normally distributed. If this was not the case, nonparametric tests were performed. One-way analysis of variance (ANOVA) was performed to analyze differences in mosquito survival rates across the three temperature conditions. Fisher’s exact tests and Chi-squared tests were used to determine whether infection, dissemination, and transmission rates differed significantly between the different conditions. Statistical comparisons of the median viral RNA loads of the three temperature conditions were performed via the Kruskal‒Wallis test, whereas the Mann‒Whitney *U* test was used for comparisons of the median viral RNA load in infection of mosquitoes that did or did not disseminate within the same temperature condition. Statistical analyses were performed using GraphPad Prism 10. *P*-values < 0.05 were considered to indicate statistical significance.

## Results

### Feeding and survival rates at different temperature conditions

**Figure 1:**
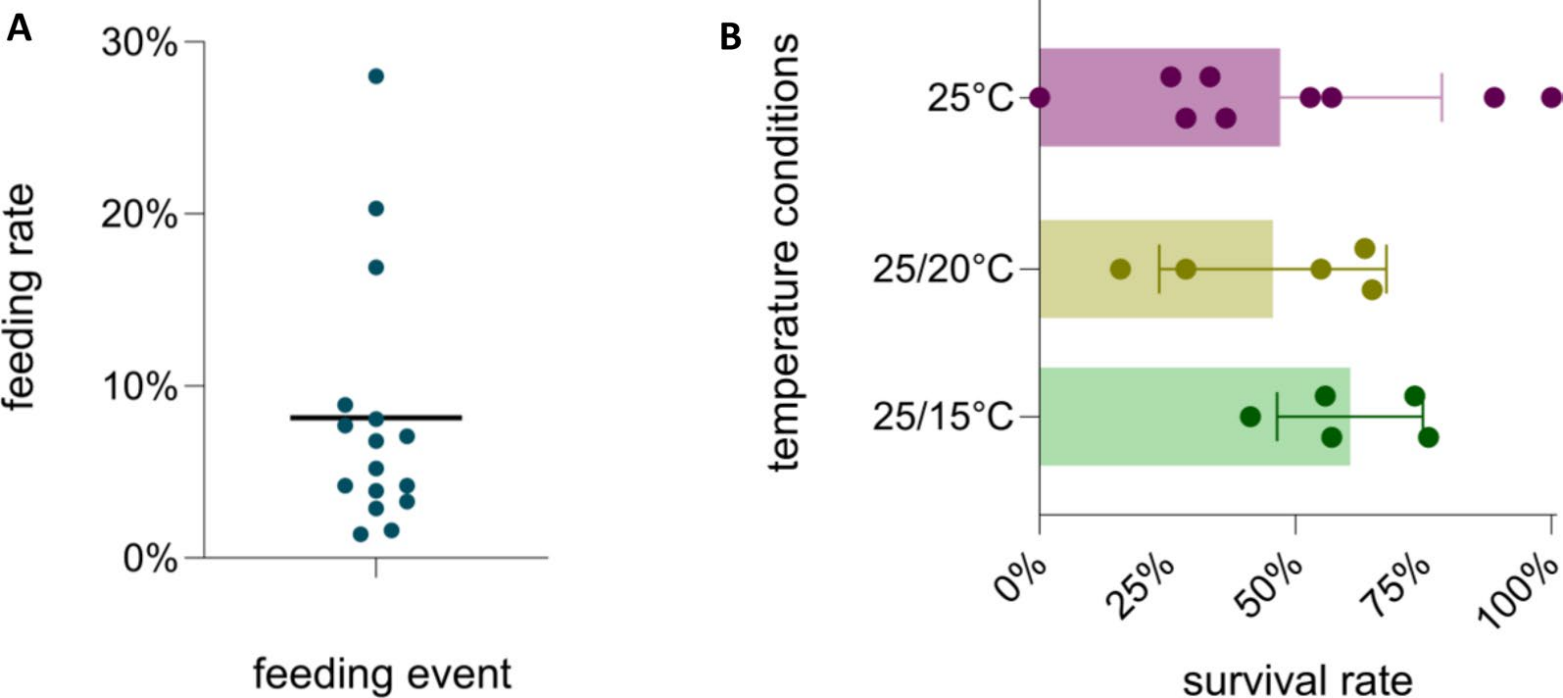
Feeding and survival rates of *An. plumbeus* in the executed feeding experiments over all tested temperature conditions. (**A**) Feeding rates of *An. plumbeus* during 19 in vitro blood-feeding events. Every dot represents a single feeding event. The horizontal line depicts the mean feeding rate. (**B**) Survival rates at 14 dpi per temperature condition: bars represent mean values with standard deviations depicted by the demarcated horizontal line, while every dot represents a single blood-feeding event

Approximately 8000 female mosquitoes were exposed to an infectious blood meal over 19 blood-feeding experiments. A mean feeding rate per blood-feeding experiment of 8.2% was observed, with a minimum feeding rate of 1.4% and a maximum of 28% (Fig. 1A). A linear regression on these data showed that the feeding rate was significantly negatively associated with the number of mosquitoes exposed to the blood meal (*β* = −0.009, *t *= −2.132, *P* = 0.049974; Additional file 1: Fig. S1), indicating that lower feeding rates were observed when more mosquitoes were exposed to the infectious blood meal. After blood-feeding, the mosquitoes were exposed to three different temperature conditions for 14 days. A total of 172 blood-fed mosquitoes survived the 14-day incubation period (62, 45, and 65 blood-fed mosquitoes at 25 °C, 25/20 °C, and 25/15 °C, respectively). The mean survival rates per temperature condition were 47%, 45.6%, and 60.7% at 25 °C, 25/20 °C, and 25/15 °C, respectively. These differences were not statistically significant (one-way ANOVA, F_(2, 16)_ = 0.8335, *P* = 0.5847) (Fig. 1B).

### WNV replication and dissemination under different temperature conditions

**Figure 2:**
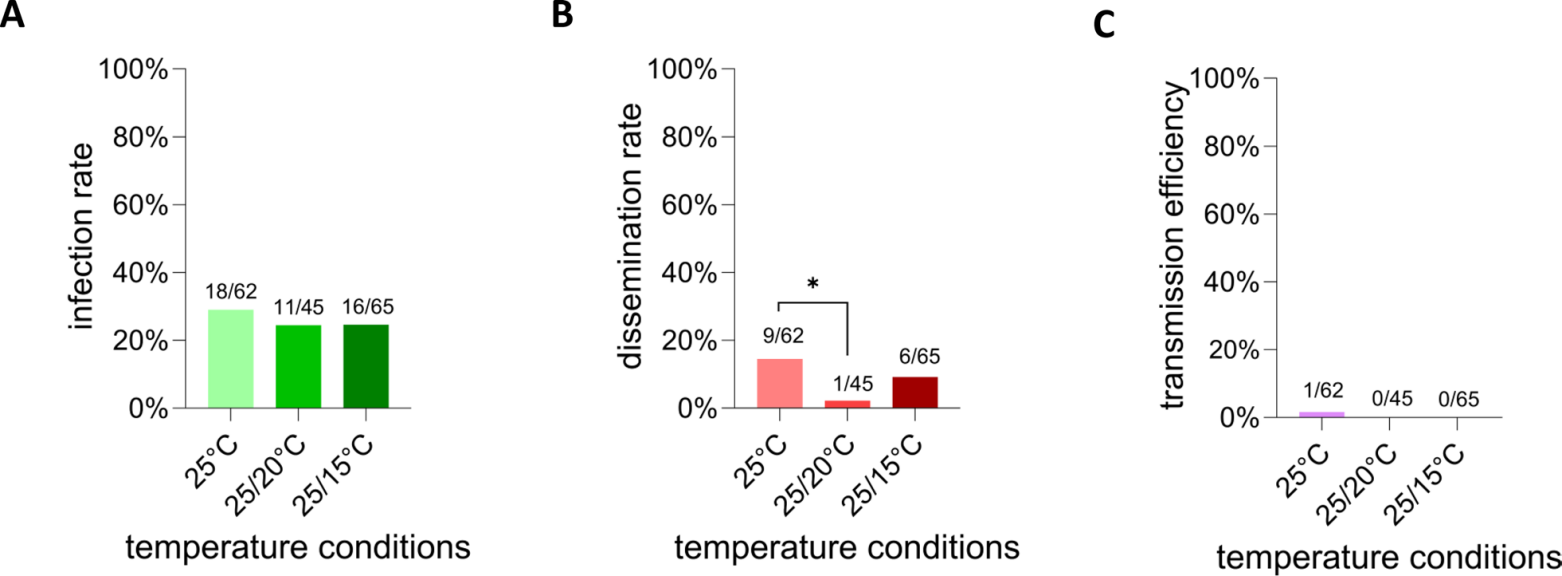
Infection rate (**A**), dissemination rate (**B**), and transmission efficiency (**C**) in *An. plumbeus* per tested temperature condition at 14 days post-blood-feeding, as determined by RT-qPCR. Absolute numbers above the bars depict in (**A**) number of WNV-positive abdomen samples in RT-qPCR/total number of mosquitoes taken an infectious blood meal, in (**B**) number of WNV-positive secondary tissues in RT-qPCR/total number of mosquitoes taken an infectious blood meal, in (**C**) number of WNV-positive saliva samples in RT-qPCR/total number of mosquitoes taken an infectious blood meal

Among the 62 engorged females surviving 14 days at 25 °C, 29% (18/62) tested positive for WNV in the abdomen/thorax via RT-qPCR, indicating successful infection of the gut, the initial site of infection in the vector. Mosquitoes incubated at a 25/20 °C day/night gradient presented an infection rate of 24.4% (11/45), whereas at the 25/15 °C day/night gradient, an infection rate of 24.6% (16/65) was found. No significant differences in infection rates among any of the three temperature conditions were observed (chi-squared test, *χ*^2^ = 0.3638, *df* = 2, *P* = 0.8337; Fig. 2A). Secondary tissues, which serve as a proxy for dissemination of the virus from the gut, were also tested for all mosquitoes. Dissemination was observed in 14.5% (9/62), 2.2% (1/45), and 9.2% (6/65) of all blood-fed mosquitoes at 25 °C, 25/20 °C, and 25/15 °C, respectively. A significant difference in dissemination rates between 25 °C and 25/20 °C was observed (Fisher’s exact test, *P *= 0.0423), whereas other statistical comparisons revealed no significant differences (Fig. 2B). Interestingly, not all mosquitoes that were positive in dissemination were also positive in their abdomens: at a constant 25 °C, five infection-negative but dissemination-positive mosquitoes were found. At 25/20 °C, one such sample was observed, and three such samples were observed at 25/15 °C. One of the 62 saliva samples at the constant 25 °C condition (1.6%), which originated from a dissemination-positive mosquito, tested positive for WNV in RT-qPCR. Under the 25/20 °C and 25/15 °C gradient conditions, no WNV-positive saliva samples were detected, resulting in 0% transmission efficiency under these conditions. No significant differences in transmission efficiency were detected (Fisher’s exact test, *P* > 0.9999) (Fig. 2C).

### Virus isolation of RT-qPCR positive samples

**Figure 3:**
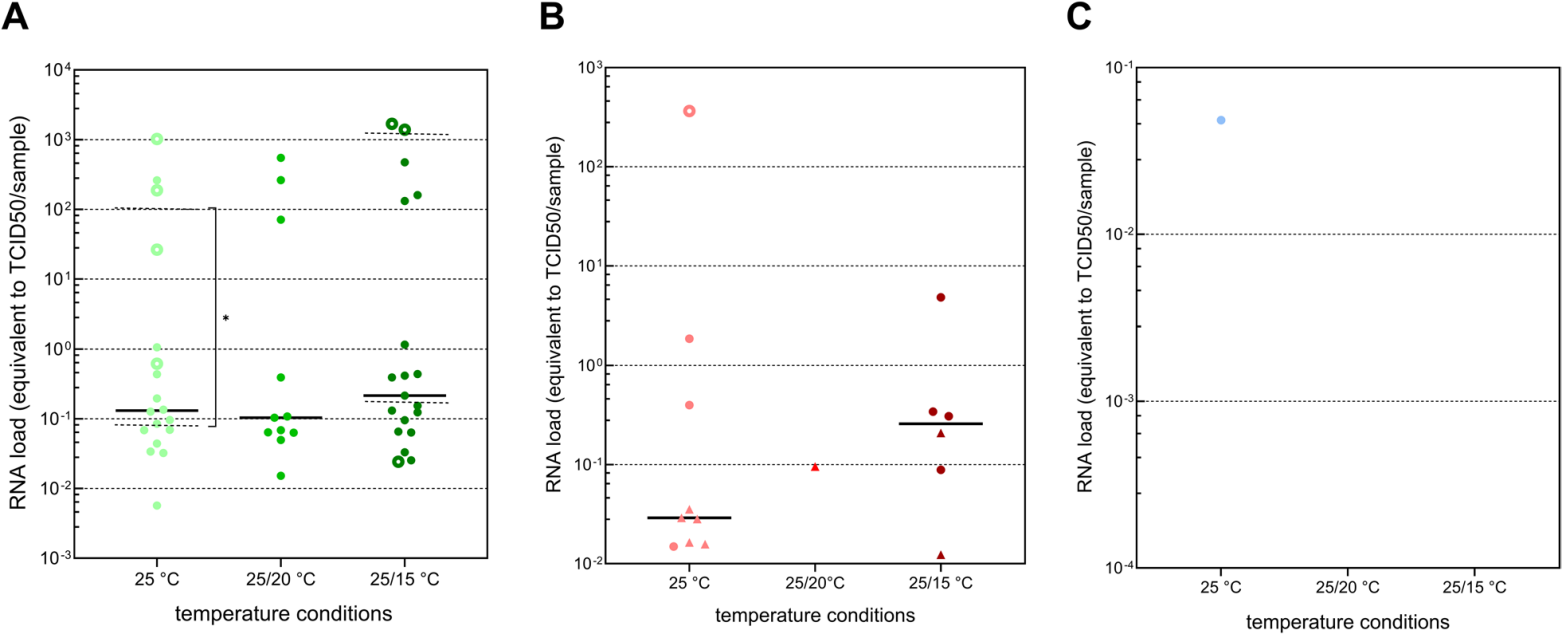
Viral RNA loads in RT-qPCR-positive infection (**A**), dissemination (**B**), and transmission samples (**C**) at the different temperature conditions. Each dot represents the viral RNA load of a single sample. In panel (**A**), filled dots represent RT-qPCR-positive infection samples that were negative in dissemination. Empty dots represent RT-qPCR-positive infection samples that were positive in dissemination. In panel (**B**), filled dots represent RT-qPCR-positive dissemination samples that were negative for transmission. Empty dots represent RT-qPCR-positive dissemination samples, which were also positive for transmission. Triangles represent samples that are positive in dissemination but negative in infection. The full horizontal line depicts the overall median value per condition. The dashed horizontal lines in (**A**) depict the median viral RNA load of infection-positive mosquitoes that were either dissemination positive or negative. (TCID50: 50% median tissue culture infectious dose)

Samples positive in RT-qPCR were tested in virus isolation to determine the presence of infectious virus. A total of 16.7%, 27.3%, and 31.3% of the RT-qPCR-positive infection samples at 25 °C, 25/20 °C, and 25/15 °C, respectively, tested positive in terms of virus isolation. A Mann–Whitney *U* statistical test showed significant differences between the viral RNA loads of infection samples that were identified as positive and negative in virus isolation in all three temperature conditions (U_(18)_ = 2, *P* = 0.0098 for 25 °C; U_(11)_ = 0, *P* = 0.0121 for 25/20 °C; U_(19)_ = 0, *P* = 0.0002 for 25/15 °C; Additional File 2: Fig. S2). None of the RT-qPCR-positive dissemination samples were positive in virus isolation (Table 1). The one RT-qPCR positive saliva sample at 25 °C contained a low viral RNA load (equivalent to < 10^–1^ TCID50; Fig. 3) and tested negative in virus isolation. Due to a technical problem of the incubator, the CO_2_ level had been below 5% for 2 days during the first passage of the isolation. The positive control samples nevertheless tested positive for WNV, indicative that the impact was limited. Insufficient saliva was available to allow for a retest.

**Table 1:** Virus isolation of infection and dissemination samples found to be positive by RT-qPCR

	Infection RT-qPCR positive samples confirmed by isolation	Dissemination RT-qPCR positive samples confirmed by isolation
25 °C	16.7% (3/18)	0% (0/9)
25/20 °C	27.3% (3/11)	0% (0/1)
25/15 °C	31.3% (5/16)	0% (0/6)

(Ratios in percentage, absolute numbers between brackets)

### Link between viral RNA load and viral dissemination

The median viral RNA load of infection-positive mosquitoes was not significantly different among the three temperature conditions (Kruskal‒Wallis test, *H* = 0.7068, *df* = 2, *P* = 0.7023). At 25 °C, a significant difference was observed between the viral RNA loads of infection-positive mosquitoes that were also positive for dissemination and those of infection-positive but dissemination-negative mosquitoes (Mann‒Whitney *U* test, U_(18)_ = 2, *Z* = −2.55, *P* = 0.0098). In the 25/15 °C temperature condition, the viral RNA loads of these two populations (infection positive – dissemination positive and infection positive – dissemination negative) did not differ significantly (Mann‒Whitney *U* test, U_(19)_ = 15, *P* = 0.3591). At 25/20 °C, no such comparison could be made, as there were no samples under these conditions that were positive for both infection and dissemination (Fig. 3A). No statistically significant differences in the median viral RNA loads of dissemination-positive mosquitoes among the three temperature conditions were observed (Kruskal‒Wallis test, *H* = 0.4828, *df* = 2, *P* = 0.8515). Only at 25 °C was there one dissemination-positive sample that was also positive for transmission (Fig. 3B). This one WNV-positive saliva sample originated from a mosquito with a high viral RNA load for dissemination (10^2.6^ TCID50 in the secondary tissue sample) (Fig. 3C). Interestingly, samples that were positive in dissemination and negative in infection were observed as well. This was the case for five samples at 25 °C, one at 25/20 °C, and three at 25/15 °C (depicted as triangles in Fig. 3B).

## Discussion

With regard to the expanding spread of WNV in Europe, and the detection of WNV cases in neighboring countries, Belgium is also increasing its preparedness for a potential introduction. In this context, evaluating the vector competence of different native mosquito species is critical for informing national surveillance and preparedness strategies. This study focused on the vector competence of *An. plumbeus*, a widespread and increasingly urbanized species in Belgium—and by extension in large parts of Europe [33]. Our goal was to assess its potential role in WNV transmission as only little is currently known on this species’ vector competence for WNV.

To obtain sufficient blood-fed mosquitoes to attain our predefined number of 50 blood-fed females at 14 dpi per condition, approximately 8000 females had to be exposed to an infectious blood meal. This high number was mostly due to the low feeding rates observed in this study. A range of different factors have been identified that can impact feeding rates, e.g., the blood source [34] and the feeding system or feeding membrane used [35, 36]. It has also been described that colonized mosquitoes tend to blood feed more easily in artificial laboratory settings then field-collected mosquitoes [16, 37]. Additionally, the low feeding rates in our study seem to be related to the high number of mosquitoes that were exposed to each blood meal. A linear regression analysis showed a significantly negative association between both parameters. Thus, when too high numbers of mosquitoes are exposed to a single blood meal, competition increases to such levels that it negatively impacts feeding rates. Therefore, it is recommended to use moderate numbers of mosquitoes per feeding event.

In this study, the vector competence of Belgian *An. plumbeus* was determined under three temperature conditions. RT-qPCR and virus isolation results showed that at a constant 25 °C, *An. plumbeus* can support WNV infection and dissemination, but transmission was observed in only 1 of the 62 blood-fed mosquitoes by RT-qPCR at 14 dpi, corresponding to a transmission efficiency of 1.6%. This transmission efficiency is low, and in line with the absence of transmission reported in a recent study by Martinet et al. [26]. Compared with reported efficiencies at constant high temperatures of *Cx. pipiens,* the main WNV vector in Europe, the transmission efficiency in our study is considered low. For example, a study on Finnish *Cx. pipiens pipiens* reported a transmission rate for WNV lineage 1 of 40% at 27 °C [38], while an Italian study reported rates ranging between 37% and 47% across various *Cx. pipiens* populations for WNV lineage 1 at 28 °C [39]. Although the higher incubation temperature may have favored greater transmission in those studies than what we observed at 25 °C, it will most likely not account for the large differences observed.

In addition to the low transmission efficiency observed at 25 °C, the viral RNA load in this one positive saliva sample was low (equivalent to 10^−1.32^ TCID50/sample). Furthermore, this sample tested negative in virus isolation. Although there is some uncertainty about this isolation result since the CO_2_ level in the incubator was too low for 2 days during the first passage of the isolation, it seems highly unlikely that a sample with such low viral RNA load could be found positive in isolation. Isolation positive samples in our study namely contained at least equivalent viral RNA loads of 10^1^ TCID50/sample (Fig. S2). Taken together, these results obtained at a constant incubation temperature indicate that Belgian *An. plumbeus* will not serve as a competent vector at 25 °C.

A key aim of this study was to evaluate how vector competence is influenced by gradient temperature conditions, which better reflect the diurnal temperature regime than a constant incubation temperature. Environmental temperature is a known abiotic factor that influences mosquito ecology, behavior, physiology, and metabolism and can thus impact vector competence and vector capacity. Therefore, we first tested how actual Belgian summer temperatures (25/15 °C) affect the vetor competence of *An. plumbeus*. Under the 25/15 °C gradient, the infection rate remained comparable to the one under the 25 °C constant condition, whereas the dissemination rate slightly decreased. No saliva samples tested positive for WNV in RT-qPCR under these gradient conditions. These results mirror findings from earlier work on the vector competence of *An. plumbeus* for JEV [29], a closely related flavivirus, which also showed loss of vector competence at 25/15 °C gradient temperatures. Global warming has an increasing impact on ectotherms, such as mosquitoes, whose internal temperature depends on the environmental temperature [40, 41]. Therefore, we also tested the vector competence at a 25/20 °C gradient. In contrast to our working hypothesis that the dissemination rate at 25/20 °C would be between those at 25 °C and 25/15 °C, the 25/20 °C condition yielded a very low dissemination rate, which was lower than at 25/15 °C. Again, no transmission was detected. The finding that there was no significant difference in the viral load in the infection samples (abdomen/thorax) between the temperature conditions, while the dissemination from the midgut to the rest of the body was significantly impaired, suggests that the temperature gradient impacts mosquito physiology. Temperature gradients probably impact the immune system of the mosquito, which is a keystone in the virus‒vector interaction. Previous research has shown that the transcriptome, including that of immune genes, is altered by environmental temperature [42]. With this knowledge in mind, we hypothesize that the mosquito’s innate immune system behaves differently under constant environmental conditions than under day/night temperature alterations. In the future this hypothesis could be further explored by investigating the expression of immune factors in infected mosquitoes at different temperatures via RNA sequencing.

In addition to temperature, other variables can impact the outcome of vector competence studies and complicate comparisons between studies. One such parameter is the incubation time, after which the vector competence is assessed. Here, we studied vector competence at 14 days post-blood-feeding. Interestingly, dissemination-positive mosquitoes that were negative for infection (five at 25 °C, one at 25/20 °C, and three at 25/15 °C), i.e., negative for the virus in the abdomen, were identified. This finding suggests that the virus had already been cleared from the gut lumen and midgut by 14 days post-blood-feeding and indicates that it would be interesting to study the viral replication kinetics in different body parts over time and to assess vector competence at earlier timepoints, such as 7 or 10 days post-blood-feeding. Additionally, variables such as the viral lineage, virus strain, viral titer, and passage should be considered when vector competence results are compared [43]. Here, a WNV lineage 1 strain was used at a representative titer of 10^5.72^ TCID50/m-L in the blood meal, but it would also be interesting to study the vector competence of a lineage 2 strain, as this is currently the predominant lineage circulating in Europe [2].

## Conclusions

This study showed that Belgian *An. plumbeus* mosquitoes will play no important role in WNV transmission upon an introduction in Belgium. At a constant temperature of 25 °C, a very low transmission efficiency was observed, with only 1 out of 62 mosquitoes testing positive for WNV in RT-qPCR. The low viral RNA load in this sample and the inability to isolate the virus furthermore indicate that Belgian *An. plumbeus* will probably not contribute to WNV transmission in Belgium. Moreover, no evidence was found to support WNV transmission by *An. plumbeus* under relevant gradient temperature conditions, as no virus could be detected in the saliva of mosquitoes incubated under a 25/20 °C or 25/15 °C temperature gradient.

## Supplementary Information

Additional file 1: Fig. S1. Linear regression analysis of feeding rate data.

Additional file 2: Fig. S2. Correlation between the detection of infectious virus and the viral load, as measured by RT-qPCR.

## Data Availability

Data supporting the main conclusions of this study are included in the manuscript.

## Abbreviations

An.: *Anopheles*
BSL-3: Biosafety level 3
CT value: Cycle threshold value
Cx.: Culex; dpi: days postinfection
JEV: Japanese encephalitis virus
RT-qPCR: Quantitative reverse transcription polymerase chain reaction
WNV: West Nile virus
