# Supplementary material for: Vector competence of Belgian Anopheles plumbeus mosquitoes for West Nile virus under different temperature conditions
Source: Parasit Vectors. 2026 Apr 3;19:213. doi: 10.1186/s13071-026-07346-9 (PMC13174012; doi:10.1186/s13071-026-07346-9)
Supplement: Supplementary file 1 — Additional file 1: Fig. S1. Linear regression analysis of feeding rate data. [file 13071_2026_7346_MOESM1_ESM.docx]

**Additional information 1**

**Figure S1: Association between number of mosquitoes exposed to blood feeding and blood feeding rate.** *An. plumbeus* mosquitoes were exposed to a WNV containing blood meal during 19 feeding events during this study. A linear regression analysis was performed to verify if there was an association between the number of mosquitoes exposed to the bloodmeal and the feeding rate, being the percentage of blood engorged females after a 1 hour feeding period. The analysis showed that the feeding rate (y-axis) is significantly negative correlated with number of exposed mosquitoes (x-axis) (β = -0.009, t = -2.132, p = 0.049974).
