# Supplementary material for: Vector competence of Belgian Anopheles plumbeus mosquitoes for West Nile virus under different temperature conditions
Source: Parasit Vectors. 2026 Apr 3;19:213. doi: 10.1186/s13071-026-07346-9 (PMC13174012; doi:10.1186/s13071-026-07346-9)
Supplement: Supplementary file 2 — Additional file 2: Fig. S2. Correlation between the detection of infectious virus and the viral load, as measured by RT-qPCR. [file 13071_2026_7346_MOESM2_ESM.docx]

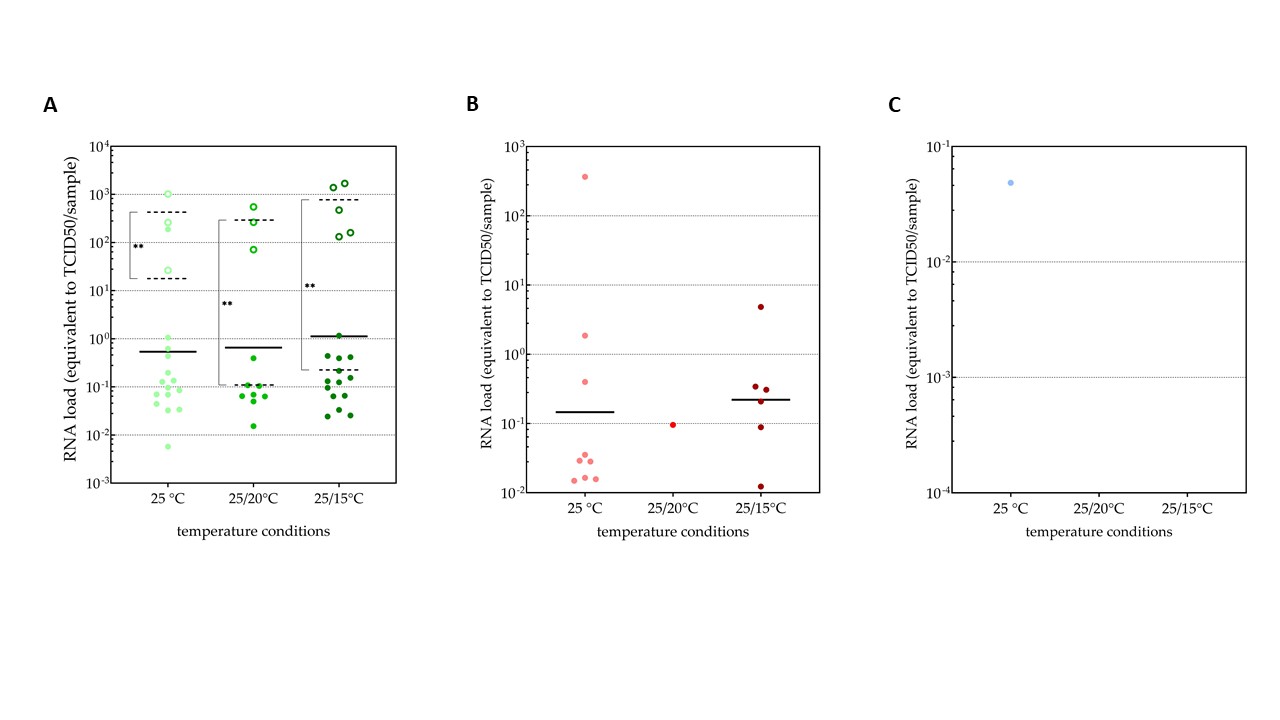
Additional information 2

**Figure S2: Correlation between detection of infectious virus and viral load, measured by RT-qPCR.** Viral RNA load of RT-qPCR positive infection (A), dissemination (B) and transmission (C) samples under the three different temperature conditions. Each dot represents the viral RNA load in a single sample. In all panels filled dots represent samples that were negative in virus isolation, whereas empty dots represent samples that were positive in virus isolation. At 25/20 °C and 25/15 °C no positive saliva samples were detected, explaining the absence of datapoints for these temperatures in (C). The full horizontal line depicts the overall mean value per condition. The dashed horizontal lines in (A) depict the mean viral RNA load of infection positive mosquitoes that were either virus isolation positive or negative.
